# Selective attrition and bias in a longitudinal health survey among survivors of a disaster

**DOI:** 10.1186/1471-2288-7-8

**Published:** 2007-02-15

**Authors:** Bellis van den Berg, Peter van der Velden, Rebecca Stellato, Linda Grievink

**Affiliations:** 1 National Institute for Public Health and the Environment (RIVM), A. van Leeuwenhoeklaan 6, 3720 BA Bilthoven, The Netherlands; 2 Institute of Risk Assessment Sciences (IRAS), Utrecht University, Yalelaan 2, 3584 CM, Utrecht, The Netherlands; 3 Institute for Psychotrauma (IvP), van Heemstraweg-west 5, 5301 PA, Zaltbommel, The Netherlands; 4 Centre for Biostatistics, Utrecht University, Padualaan 14, 3584 CH, Utrecht, The Netherlands

## Abstract

**Background:**

Little is known about the response mechanisms among survivors of disasters. We studied the selective attrition and possible bias in a longitudinal study among survivors of a fireworks disaster.

**Methods:**

Survivors completed a questionnaire three weeks (wave 1), 18 months (wave 2) and four years post-disaster (wave 3). Demographic characteristics, disaster-related factors and health problems at wave 1 were compared between respondents and non-respondents at the follow-up surveys. Possible bias as a result of selective response was examined by comparing prevalence estimates resulting from multiple imputation and from complete case analysis. Analysis were stratified according to ethnic background (native Dutch and immigrant survivors).

**Results:**

Among both native Dutch and immigrant survivors, female survivors and survivors in the age categories 25–44 and 45–64 years old were more likely to respond to the follow-up surveys. In general, disasters exposure did not differ between respondents and non-respondents at follow-up. Response at follow-up differed between native Dutch and non-western immigrant survivors. For example, native Dutch who responded only to wave 1 reported more depressive feelings at wave 1 (59.7%; 95% CI 51.2–68.2) than Dutch survivors who responded to all three waves (45.4%; 95% CI 41.6–49.2, *p *< 0.05). Immigrants who responded only to wave 1 had fewer health problems three weeks post-disaster such as depressive feelings (M = 69.3%; 95% CI 60.9–77.6) and intrusions and avoidance reactions (82.7%; 95% CI 75.8–89.5) than immigrants who responded to all three waves (respectively 89.9%; 95% CI 83.4–96.9 and 96.3%; 95% CI 92.3–100, *p *< .01). Among Dutch survivors, the imputed prevalence estimates of wave 3 health problems tended to be higher than the complete case estimates. The imputed prevalence estimates of wave 3 health problems among immigrants were either unaffected or somewhat lower than the complete case estimates.

**Conclusion:**

Our results indicate that despite selective response, the complete case prevalence estimates were only somewhat biased. Future studies, both among survivors of disasters and among the general population, should not only examine selective response, but should also investigate whether selective response has biased the complete case prevalence estimates of health problems by using statistical techniques such as multiple imputation.

## Background

Epidemiologic studies after disasters have shown elevated levels of health problems among survivors such as post-traumatic stress disorder (PTSD), depression and physical symptoms [1-3]. The majority of the disaster studies have been cross-sectional, and although cross-sectional studies are useful for assessing the public health burden of the disaster, they do not give insight into the course of the health consequences and the health needs of survivors at different times post-disaster.

Since relatively little is known about the course of health problems among survivors of disasters, more longitudinal studies are needed [1,2,4]. However, attrition is a main methodological problem in longitudinal studies. A common approach for handling attrition is to delete observations with missing values, but this complete case analysis can result in a substantial loss of power. In addition, if respondents systematically differ from non-respondents, deleting incomplete observations might introduce bias in the prevalence estimates of the health problems [5,6]. A good way to deal with missing data and to overcome possible selection bias in the prevalence estimates is to conduct multiple imputation [6,7]. This technique fills in various values for each missing data point based on a statistical model. Because the missing values are drawn from a distribution, there will be a range of values imputed for each missing value, with variation appropriately reflecting the uncertainty about that value. Using this technique, it can be estimated what the prevalence of the outcomes of interest would have been if there had been no (systematic) attrition in the longitudinal study.

Evidence concerning selective response among survivors of disasters is conflicting. Some studies have shown that non-respondents are more likely to be male, single and to have a low socioeconomic status [8-10], while other studies did not observe such an association [11-15]. Little is known about the association between the level of disaster exposure and non-response at follow-up. One may speculate that survivors who were highly affected by the disaster or who had high levels of post-disaster distress would be more motivated to participate at the follow-up of a health survey than survivors who were less affected. On the other hand, it can be hypothesized that highly exposed or distressed survivors would be less likely to respond because they do not want to be reminded of the stressful event. Several studies have found that depression, distress and symptoms of PTSD at baseline were associated with non-response at follow-up [8,16,17] while other studies found no association between baseline distress and non-response at follow-up [12-15]. In addition, the determinants of response at follow-up might also differ between groups of survivors. We recently observed that baseline health problems were associated with response among immigrant survivors and with non-response among native Dutch survivors at wave 2 of a study after a fireworks disaster in the Netherlands [18]. The information from these disaster studies is, however, not sufficient to understand the response mechanisms of survivors of disasters. Furthermore, none of the previous studies after disasters have examined whether selective response biased the prevalence estimates of the health problems among survivors.

Since attrition will most likely occur in future longitudinal studies after disasters, more insight into the response mechanisms among survivors and possible bias resulting from selective response is desirable. In the present longitudinal study after a fireworks disaster in the Netherlands, we examine the selective response among survivors at the follow-up surveys. In addition, we study whether possible selective response had biased the prevalence estimates of health problems among survivors at wave 3 by comparing the estimates resulting from multiple imputation with estimates resulting from complete case analysis.

## Methods

### Background

On May 13 2000, a fireworks depot exploded in a residential area in the city of Enschede, the Netherlands. As a result of the explosion and subsequent fire, 23 persons were killed, more than 900 people were injured, and about 1,200 people were forced to relocate because their houses were destroyed or severely damaged. The Dutch government declared this a national disaster and started a longitudinal study into the health consequences of the disaster.

### Study design

The first survey was performed 2.5 to 3.5 weeks post-disaster (wave 1). In total, 4,456 adult residents were living in the area that was designated by the municipality as the official disaster area. All residents of this area were invited to participate in the health survey by means of announcements in the local media and letters.

Approximately 18 months after the disaster, from November 2001 through January 2002, a second survey was conducted (wave 2). All participants at wave 1 who had given informed consent for future contact received an announcement letter. To stimulate participation, survivors were telephoned. If the survivor agreed to participate, a questionnaire in the preferred language (Dutch, English, German or Turkish) was sent, together with a gift voucher, to their home address. Survivors who did not return the questionnaire within three weeks were reminded by phone or by letter when the person could not be reached by phone.

In January through March 2004, nearly four years post-disaster, wave 3 of the longitudinal study was performed. All participants at wave 1 of the health survey who had given informed consent for future contact, and were not lost to follow-up, received an announcement letter. Participation was stimulated by means of telephone calls and home visits. If the questionnaire was not returned within three weeks, the respondents were reminded by phone or by letter. Details of the study population and the health problems of survivors at the different waves of the study have been described elsewhere [18-22].

### Measures

We selected the following demographic variables to examine possible selective response: sex; age; educational level; employment status (having a paid job), and marital status (single).

To examine whether respondents and non-respondents at waves 2 and 3 had different disaster-related experiences, the following disaster-related factors were selected: injury (requiring medical treatment) sustained as a result of the disaster; the loss of loved ones (family, colleagues, friends); relocation due to severely damaged or destroyed house; whether survivors had experienced intense anxiety during the disaster and whether survivors had seen frightening things during the disaster.

We compared health problems at wave 1 between respondents and non-respondents at the follow-up surveys to study possible selective response. We used the Dutch versions of various validated instruments to measure health problems. Feelings of depression and anxiety were measured by the symptom check list (SCL-90) [23,24]. We dichotomized the scales into 'very high' and 'high' versus 'above average', 'average' and below 'average', according to established references for the healthy Dutch population [24]. The impact of event scale (IES) [25-27] was used to measure intrusions and avoidance reactions which serve as an indication for a clinical level of PTSD. Consistent with Carr et al., Basoglu et al. and others, survivors with an overall score above 25 were considered as having symptoms of PTSD [28,29]. We used a questionnaire into subjective health complaints (VOEG) [30] to measure 13 physical symptoms such headache and fatigue. In this study, we used a cut-off of six or more symptoms which is one standard deviation above the reference mean. Sleeping difficulties were measured by the Groninger Sleep Quality Scale [31]; survivors with a score above 4 were defined as having severe sleeping difficulties. The RAND-36 was used to measure different aspects of functional status [32]. To examine selective response, four sub-scales were used; social functioning, physical role limitations, emotional role limitations and general health. Scores on the sub-scales were dichotomized using the cut-off scores resulting from a national study in the Netherlands; in this study cut-off scores were based on the mean score minus one standard deviation [33]. We used the scales described above as the outcomes of interest to examine whether possible selective response had biased the prevalence estimates of the health problems at wave 3.

### Statistical analyses

Because the response mechanisms between waves 1 and 2 were found to be different for native Dutch survivors and non-western immigrant survivors [18], we stratified the analysis according to ethnic background (native Dutch and immigrant status) in order to study selective response and possible biased prevalence estimates. We defined a non-western immigrant as either a respondent who was born in a non-western country of whom at least one parent was also born in a non-western country, or a respondent whose parents were both born in a non-western country. Most of the non-western immigrants were of Turkish origin (44.7%), followed by immigrants of Moroccan origin (14.0%).

We compared demographic characteristics, disaster-related experiences and wave 1 health problems for respondents and non-respondents at waves 2 and 3 in order to study possible selective response by performing Chi-square tests for categorical variables and ANOVA for continuous variables.

We used multiple imputation (MI) to study the effect of possible selective response on the prevalence estimates of the wave 3 health problems [5]. MI assumes that the missing data are "missing at random"; in other words, the missingness is not related to factors that were not measured in this study. Multiple imputations were performed with the Markov chain Monte Carlo (MCMC) simulation in SAS version 9.1 using the 'MI' procedure. The method imputes plausible values for the missing data using correlations between observed variables [5]. For that reason we included the health problems of interest (described above) from all three waves in the imputation model. In addition, other relevant predictor variables of these health problems were selected; sex; age; educational level; immigrant status; employment status; language; cigarette smoking; alcohol use; sustained injury due to the disaster; relocation; intense anxiety and having seen frightening things during the disaster. Since the power of the model increases when additional data other than the variables of interest are used [6], other important health-related variables were selected: the somatization, hostility and interpersonal sensitivity sub-scales of the SCL-90 [23,24], the use of sedatives and the presence of chronic diseases among survivors. These variables were measured at all three waves. Peritraumatic dissociation [34] (measured only at wave 1), and optimism [35], the distrust sub-scale of the search for meaning scale [36], and the pain, vitality and mental health sub-scales of the RAND-36 [33] (all measured at waves 2 and 3) were also included in the imputation model. In addition, we included a variable for the approach of participants at wave 3 of the health survey (no contact, telephone contact or face to face).

We used one MI model for both the native Dutch and immigrant survivors because separate analysis results in a substantial loss of power and such an analysis assumes that there is a difference between native Dutch and immigrant for each variable in the MI model. Because some determinants of response differed between native Dutch and immigrant survivors, interaction terms for immigrant status and gender, educational level, marital status, relocation, injury self, lost a loved one, intense anxiety, saw frightening things, feelings of depression, feelings of anxiety, physical symptoms, sleeping problems, social functioning, physical and emotional role limitations, and general health (measured at the three waves) were entered into the model. We did not dichotomize the variables entered in the model, instead linear effects were used in the MI model.

We generated five datasets that were analyzed separately. The results were combined using the 'MIANALYZE' procedure in SAS, in order to produce valid confidence intervals. Finally, we compared the imputed prevalence estimates of wave 3 health problems with the prevalence estimates resulting from complete case analysis for native Dutch and immigrant survivors separately.

## Results

### Selective response at waves 2 and 3 among native Dutch survivors

**Figure 1:**
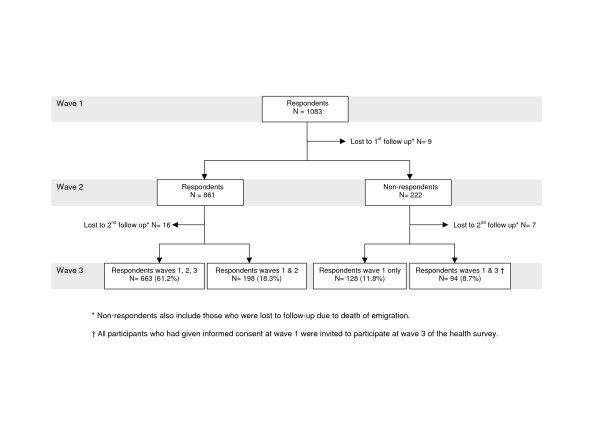
Flow chart response native Dutch survivors.

In total, 1,083 native Dutch survivors completed the questionnaire at wave 1 (figure 1). Of these survivors, 663 (61.2%) participated at all three waves of the longitudinal study. Three other response groups can be distinguished: 128 survivors (11.8%) who responded to wave 1 only; 198 survivors who responded to waves 1 and 2 (18.3%); and 94 survivors who responded to waves 1 and 3 (8.7%).

Demographic characteristics of native Dutch respondents and non-respondents at the three waves are shown in table 1. There were some demographic differences between respondents and non-respondents at the follow-up surveys; men were more likely to respond to wave 1 only, while native Dutch women were more likely to respond to all three waves of the study. Those survivors who responded to waves 1 and 3 but not to wave 2 were younger than the other response groups. Native Dutch survivors who responded only to wave 1 or to waves 1 and 2 had a somewhat lower educational level than survivors who responded to wave 3, however this difference was not statistically significant. Finally, respondents who responded to all three waves were less likely to live alone than those who responded to waves 1 and 2.

**Table 1:** Demographic characteristics of native Dutch respondents and non-respondents at the three waves of the longitudinal study*

	Respondents at Wave 1 only (N = 128)	Respondents at Waves 1 & 2 (N = 198)	Respondents at Waves 1 & 3 (N = 94)	Respondents at Waves 1, 2, 3 (N = 663)	*p *value
	% (95% CI)	% (95% CI)	% (95% CI)	% (95% CI)	
Male	59.8 (51.2–68.3)	49.0 (42.0–56.0)	48.9 (38.7–59.0)	42.8 (39.0–46.6)	0.004
Age					
18 – 24	16.4 (9.9–22.8)	9.6 (5.5–13.7)	19.2 (11.1–27.2)	10.4 (8.1–12.7)	0.004
25 – 44	43.0 (34.3–51.6)	43.9 (37.0–50.8)	53.2 (43.0–63.3)	43.9 (40.1–47.7)	
45 – 64	26.6 (18.9–34.3)	30.3 (23.9–36.7)	22.3 (13.8–30.7)	35.0 (31.4–38.6)	
65+	14.1 (8.0–20.1)	16.2 (11.1–21.3)	5.3 (0.7–9.8)	10.7 (8.4–13.1)	
Age, Mean	42.0 (39.4–44.9)	45.1 (42.7–47.4)	37.3 (34.4–40.2)	43.5 (42.4–44.7)	0.0005
Education					
Primary school	14.9 (8.7–21.1)	14.5 (9.6–19.4)	8.0 (2.4–13.5)	10.4 (8.1–12.7)	0.4
Junior high	37.2 (28.7–45.6)	32.3 (25.8–38.8)	8.0 (2.4–13.5)	10.4 (8.1–12.7)	
Senior high/professional	34.7 (26.4–43.0)	33.9 (27.3–40.5)	30.7 (21.3–40.0)	33.7 (30.1–37.3)	
High professional/university	13.2 (7.3–19.1)	19.4 (13.9–24.9)	25.0 (16.1–33.8)	21.6 (18.5–24.7)	
Paid job	62.0 (53.5–70.4)	57.4 (50.5–64.3)	75.3 (66.5–84.0)	64.0 (60.4–67.7)	0.03
Single	24.4 (16.9–31.8)	26.5 (20.4–32.6)	20.0 (11.8–28.1)	17.8 (14.9–20.7)	0.04

* Groups of respondents are exclusive; Anova was used for continuous variables and X^2^-tests for categorical variables

Table 2 shows the associations between response and disaster-related experiences and wave 1 health problems for the different native Dutch response groups. There were no clear differences in disaster exposure among the different groups, except for a lower percentage of injured survivors among those who responded to waves 1 and 2 but not to wave 3 compared to survivors who responded to all three waves. Native Dutch survivors who participated only at wave 1 were more likely to report a high level of feelings of depression (59.7%; 95% CI 51.2–68.2) compared to those who responded in all three waves (45.4%; 95% CI 41.6–49.2). Also, wave 1 only respondents tended to have somewhat more problems with social functioning at wave 1 compared to the other response groups, though this difference was not statistically significant. Overall, survivors who responded to waves 1 and 2 but not to wave 3 seemed to have lower levels of health problems three weeks post-disaster compared to the other response groups. Finally, the wave 1 health problems among respondents at waves 1 and 3 tended to be similar to all-wave respondents.

**Table 2:** Disaster exposure and health problems three weeks post-disaster among native Dutch respondents and non-respondents at the three waves of the longitudinal study*

	Respondents at wave 1 only (N = 128)	Respondents at waves 1 & 2 (N = 198)	Respondents at waves 1 & 3 (N = 94)	Respondents at waves 1, 2, 3 (N = 663)	*p *value
	% (95% CI)	% (95% CI)	% (95% CI)	% (95% CI)	
**Disaster exposure**					
Relocated	25.4 (17.9–32.9)	16.2 (11.1–21.3)	20.4 (12.2–28.6)	20.1 (17.1–23.2)	0.3
Injury self	6.3 (2.1–10.5)	1.5 (0.0–3.2)	5.3 (0.7–9.8)	8.3 (6.2–10.4)	.009
Lost a loved one	6.3 (2.1–10.5)	4.6 (1.7–7.5)	4.3 (0.2–8.4)	6.1 (4.3–7.9)	0.8
Intense anxiety	59.6 (51.1–68.1)	53.0 (46.1–60.0)	47.9 (37.7–58.0)	60.5 (56.8–64.2)	0.05
Saw frightening things	24.2 (16.8–31.6)	19.7 (14.2–25.2)	28.7 (19.4–37.8)	24.7 (21.4–28.0)	0.3
**Health problems at wave 1**					
Depressive feelings (high)	59.7 (51.2–68.2)	44.8 (37.9–51.7)	50.0 (39.8–60.0)	45.4 (41.6–49.2)	0.04
Feelings of anxiety (high)	50.0 (41.3–58.7)	38.9 (32.1–45.7)	46.7 (36.5–56.8)	41.9 (38.1–45.7)	0.2
Intrusion and avoidance (high)	70.1 (62.2–78.0)	67.9 (61.4–74.4)	68.5 (59.0–77.9)	70.7 (67.2–74.2)	0.9
Physical symptoms (high)	48.8 (40.1–57.5)	42.2 (35.5–49.1)	43.6 (33.5–53.6)	46.7 (42.9–50.5)	0.6
Sleeping problems (high)	41.6 (33.1–50.1)	38.5 (31.7–45.3)	41.8 (31.7–51.8)	44.6 (40.8–48.4)	0.5
Poor social functioning	52.4 (43.8–61.1)	37.1 (30.4–43.8)	41.9 (31.8–51.9)	42.6 (38.8–46.4)	0.06
Physical role limitations	56.1 (47.5–64.7)	47.7 (40.7–54.7)	53.2 (43.0–63.3)	59.1 (55.4–62.8)	0.2
Emotional role limitations	72.2 (64.4–80.0)	61.4 (54.6–68.2)	69.7 (60.3–79.0)	78.8 (75.7–81.9)	0.0001
Poor general health	20.2 (13.3–27.2)	17.9 (12.6–23.2)	16.5 (8.9–24.0)	17.7 (14.8–20.6)	0.9

* Groups of respondents are exclusive

### Selective response at waves 2 and 3 among survivors of non-western origin

**Figure 2:**
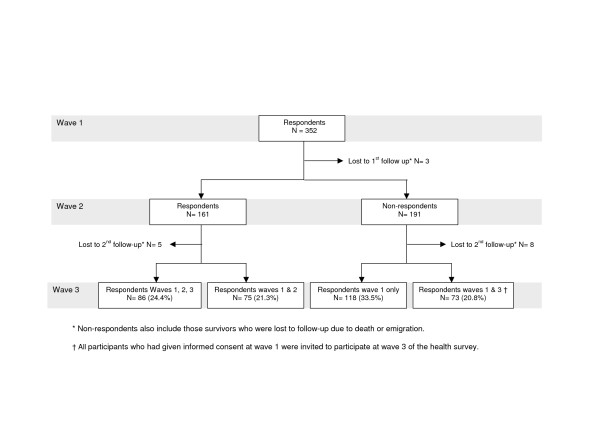
Flow chart response survivors of non-western origin.

At wave 1 of the health survey, 352 survivors of non-western origin participated (figure 2). Of this group, only 86 (24.4%) responded to all three waves; 118 immigrant survivors (33.5%) responded to wave 1 only, 75 immigrants (21.3%) responded to waves 1 and 2, and 73 immigrants (20.8%) responded to waves 1 and 3.

Male survivors of non-western origin tended to respond to wave 1 only, while female immigrant survivors were somewhat more likely to respond also to wave 3, but this difference was not statistically significant. In addition, immigrants who responded to waves 1 and 2 were somewhat older than respondents at wave 1 only and respondents at waves 1 and 3 (table 3).

**Table 3:** Demographic characteristics of respondents and non-respondents of non-western origin at the three waves of the longitudinal study*

	Respondents at wave 1 only (N = 118)	Respondents at waves 1 & 2 (N = 75)	Respondents at waves 1 & 3 (N = 73)	Respondents at waves 1, 2, 3 (N = 86)	*p *value
	% (95% CI)	% (95% CI)	% (95% CI)	% (95% CI)	
Male	53.4 (44.3–62.4)	48.0 (36.5–59.3)	45.2 (33.6–56.6)	45.4 (34.7–55.9)	0.6
Age					
18 – 24	20.5 (13.1–27.8)	12.2 (4.7–19.6)	17.8 (8.9–26.6)	8.1 (2.2–13.9)	0.03
25 – 44	55.6 (46.5–64.6)	43.2 (31.8–54.4)	57.5 (46.0–68.8)	60.5 (50.0–70.8)	
45 – 64	22.2 (14.6–29.7)	40.5 (29.2–51.6)	21.9 (12.3–31.4)	31.4 (21.4–41.2)	
65+	1.7 (0.0–4.0)	4.1 (0.0–8.6)	2.7 (0.0–6.4)	0.0	
Age, Mean	35.9 (33.5–38.4)	41.1 (37.9–44.2)	36.4 (33.4–39.4)	38.5 (36.1–40.8)	0.04
Education					
Primary school	39.8 (30.9–48.6)	52.1 (40.6–63.4)	43.1 (31.6–54.5)	38.3 (27.9–48.6)	0.8
Junior high	23.9 (16.1–31.6)	21.1 (11.7–30.3)	23.5 (13.6–33.2)	22.2 (13.3–31.0)	
Senior high/professional	29.2 (20.9–37.4)	22.5 (12.9–32.0)	23.6 (13.7–33.3)	29.6 (19.8–39.3)	
High professional/university	7.1 (2.4–11.7)	4.2 (0.0–8.7)	9.7 (2.8–16.5)	9.9 (3.5–16.2)	
Paid job	41.4 (32.4–50.3)	40.6 (29.3–51.7)	46.0 (34.4–57.4)	39.2 (28.7–49.5)	0.9
Single	23.3 (15.6–30.9)	15.3 (7.0–23.5)	17.1 (8.3–25.7)	12.9 (5.7–20.0)	0.3

* Groups of respondents are exclusive; Anova was used for continuous variables and X^2^-tests for categorical variables

Non-western immigrants who did not respond to all three waves tended to have a somewhat lower level of exposure to the disaster than survivors who responded to all three waves of the health survey, although this was not true for personal injury (table 4). In addition, immigrant survivors who responded to wave 1 only as well as those who responded to waves 1 and 3 had a lower level of health problems three weeks post-disaster compared to survivors who responded to all three waves. For example, those who responded to wave 1 only and those who responded to waves 1 and 3 had a lower level of intrusions and avoidance reactions, physical symptoms, and sleeping problems.

**Table 4:** Disaster exposure and health problems three weeks post-disaster among respondents and non-respondents of non-western origin at the three waves of the longitudinal study*

	Respondents at wave 1 only (N = 118)	Respondents at waves 1 & 2 (N = 75)	Respondents at waves 1 & 3 (N = 73)	Respondents at waves 1, 2, 3 (N = 86)	*p *value
	% (95% CI)	% (95% CI)	% (95% CI)	% (95% CI)	
**Disaster exposure**					
Relocated	21.1 (13.7–28.5)	34.8 (23.9–45.6)	32.8 (21.9–43.6)	38.6 (28.2–48.9)	0.05
Injury self	8.2 (3.2–13.2)	8.2 (1.9–14.4)	13.0 (5.2–20.7)	7.0 (1.5–12.4)	0.6
Lost a loved one	10.9 (5.2–16.5)	4.1 (0.0–8.6)	8.7 (2.1–15.2)	9.3 (3.1–15.4)	0.4
Intense anxiety	76.3 (68.6–84.0)	72.0 (61.7–82.2)	71.2 (60.7–81.6)	80.2 (71.6–88.6)	0.5
Saw frightening things	58.5 (49.5–67.4)	64.0 (53.0–74.9)	56.2 (44.6–67.6)	68.6 (58.6–78.4)	0.3
**Health at wave 1**					
Depressive feelings (high)	69.3 (60.9–77.6)	84.6 (76.3–92.8)	74.6 (64.5–84.6)	89.9 (83.4–96.3)	0.004
Feelings of anxiety (high)	66.7 (58.1–75.2)	80.3 (71.2–89.3)	74.6 (64.5–84.6)	81.5 (73.2–89.7)	0.09
Intrusion and avoidance (high)	82.7 (75.8–89.5)	93.9 (88.4–99.3)	82.4 (73.5–91.1)	96.3 (92.3–100.0)	0.006
Physical symptoms (high)	58.7 (49.7–67.6)	70.3 (59.8–80.6)	52.9 (41.0–64.1)	76.5 (67.4–85.5)	0.007
Sleeping problems (high)	61.8 (52.9–70.6)	83.6 (75.1–92.0)	59.4 (48.0–70.7)	85.9 (78.4–93.3)	0.008
Poor social functioning	69.0 (60.6–77.3)	71.8 (61.5–82.0)	72.5 (62.1–82.7)	76.7 (67.6–85.6)	0.7
Physical role limitations	71.9 (63.7–80.0)	73.2 (63.0–83.2)	67.4 (56.5–78.2)	81.2 (72.8–89.5)	0.4
Emotional role limitations	73.6 (65.6–81.6)	87.5 (79.9–95.0)	83.7 (75.1–92.2)	84.1 (76.3–91.8)	0.2
Poor general health	48.0 (38.9–57.0)	66.7 (55.9–77.4)	60.3 (48.9–71.5)	75.6 (66.4–84.7)	0.002

* Groups of respondents are exclusive.

### Comparison between imputed and complete case prevalence estimates of wave 3 health problems among native Dutch survivors

**Figure 3:**
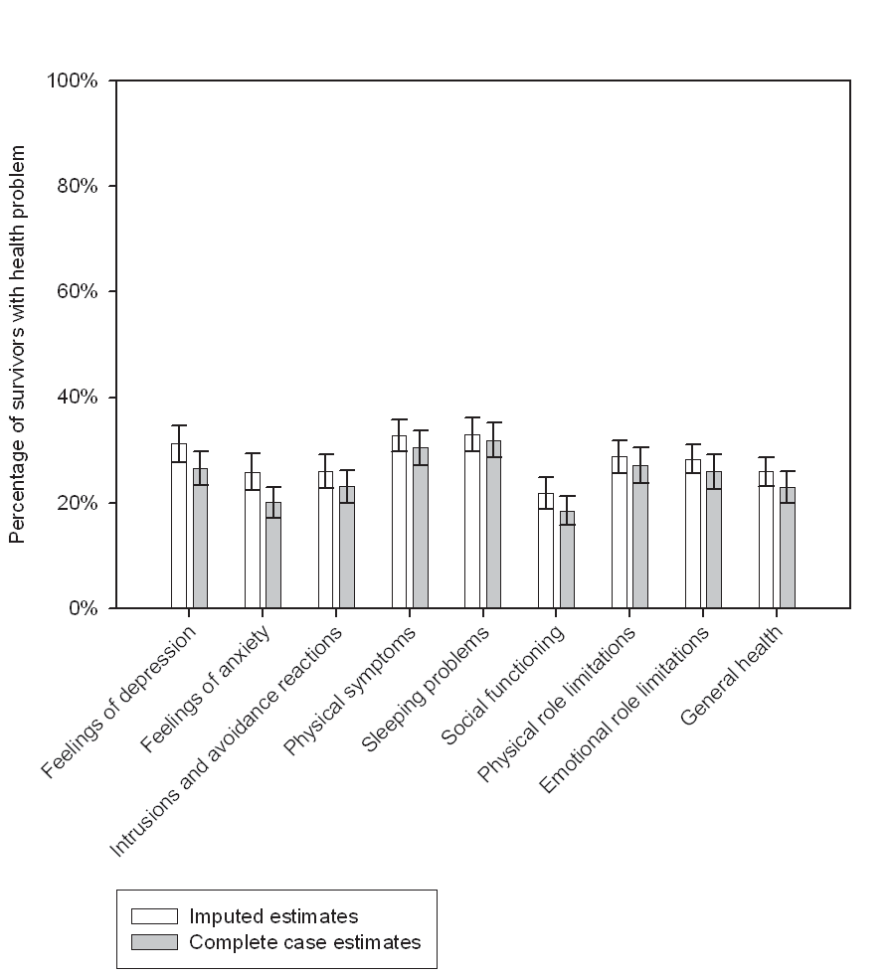
Imputed and complete case prevalence estimates of health problems at wave 3 among native Dutch survivors.

Figure 3 shows the imputed and complete case prevalence estimates of wave 3 health problems among native Dutch survivors. The imputed prevalence estimates were systematically higher than the prevalence estimates resulting from the complete case analyses. The most notable difference between the imputed and complete case estimates was found for feelings of anxiety (prevalence 25.3%; 95% CI, 22.4–28.5 and prevalence 20.1%; 95% CI, 17.2–23.0 respectively).

### Comparison between imputed and complete case prevalence estimates of wave 3 health problems among survivors of non-western origin

**Figure 4:**
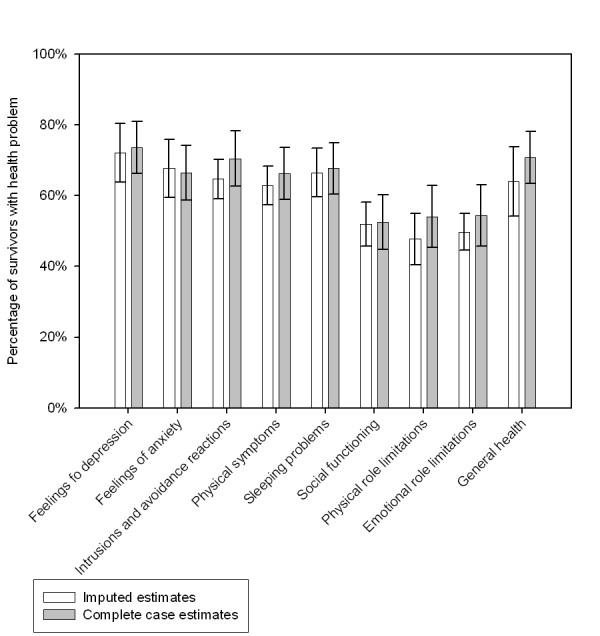
Imputed and complete case prevalence estimates of health problems at wave 3 among survivors of non-western origin.

The prevalence estimates of the health problems at wave 3 among non-western survivors resulting from multiple imputation and complete case analysis are shown in figure 4. The imputed estimates for depressive feelings, feelings of anxiety, sleeping problems and social functioning hardly differed from the estimates resulting from complete case analysis. The imputed prevalence estimates of intrusions and avoidance reactions, physical symptoms, physical and emotional role limitations and general health tended to be lower than the complete case estimates.

## Discussion

In this study among survivors of a fireworks disaster, selective response occurred at the two follow-up surveys. We examined whether selective response had biased the prevalence estimates of the wave 3 health problems by comparing prevalence estimates resulting from complete case analysis and estimates resulting from multiple imputation. The complete case prevalence estimates of the wave 3 health problems were only somewhat biased, and the direction differed between the native Dutch survivors and immigrant survivors.

Similar to other studies after disasters, non-respondents at follow-up were more likely to be male [8-10]. In agreement with two longitudinal studies after disasters, we did not find an association between damaged or destroyed house or property loss and non-response at follow-up [10,14]. In addition, we did not find clear associations between response at follow-up and other disaster-related experiences, such as the loss of a loved one and intense anxiety.

In this study, we found an association between health problems at wave 1 and response at waves 2 and 3. Some previous studies also showed an association between health problems at baseline and non-response at follow-up [8,16,17]. However, the response mechanisms in this study differed between native Dutch survivors and survivors of non-western origin. Among native Dutch survivors, health problems at wave 1 tended to be associated with non-response at follow-up (waves 2 and 3). In contrast, among immigrant survivors, health problems at wave 1 were associated with response at follow-up. These different response mechanisms between native Dutch and immigrant survivors were also found in a recent study among the survivors of the fireworks disaster in which determinants for response at wave 2 of the health survey were examined [18]. It can be speculated that immigrant survivors of the fireworks disaster were not accustomed to participation in a health survey and believed that completion of the questionnaire was not meaningful in the absence of health problems. Although the underlying reasons remain unclear, different response mechanisms among ethnic groups have also been found in a longitudinal general population study by Psaty et al [37]. In their study among whites and non-whites in the USA, poor health status was associated with non-response among whites and with response at follow-up among non-whites.

In this study we allowed survivors who participated at wave 1 but not at wave 2 to re-enter the study at wave 3. At wave 3, all eligible survivors were strongly motivated to participate; all survivors were stimulated to participate by means of telephone calls. In addition, eligible survivors for whom the telephone number was unknown and all immigrant survivors were visited at home. Survivors who re-entered the study at wave 3 differed from survivors who did not (wave 1 only). For example, among both native Dutch and immigrant survivors, those who re-entered the study at wave 3 were somewhat less likely to be male, were somewhat more likely to have a paid job and were somewhat less likely to be single. This group of respondents indirectly provides insight into non-response at wave 2 as well as additional information that is useful when performing MI.

The different response mechanisms among native Dutch and immigrant survivors were also confirmed after multiple imputation of the missing values. Among native Dutch, the imputed estimates of the wave 3 health problems tended to be higher than the complete case estimates. In contrast, the imputed prevalence estimates among immigrant survivors tended to be somewhat lower than the estimates of health problems at wave 3 resulting from complete case analysis. Additional analyses showed that the differences between imputed and complete case estimates of wave 2 health problems were similar to the differences between the complete case and imputed prevalence estimates of wave 3 health problems (data not shown). This confirms the robustness of our findings, since both native Dutch and immigrant survivors have similar response mechanisms from wave 1 to wave 2 and from wave 2 to 3.

We could not demonstrate very large differences between the imputed and complete case estimates of the health problems at wave 3. This result was unexpected since the selective response at the follow-up surveys would suggest prevalence estimates that were more strongly biased. While these results are reassuring, we can not exclude that some of the prevalence estimates were more biased than our results indicate. First, the lack of biased prevalence estimates at wave 3 might be due to weak associations between the predictor and outcome variables in the imputation model. Second, it is possible that other variables, not included in the model, were more important predictors of response and that missing data were not missing at random. However, we included all variables in the imputation model that were likely to be related to response in a health survey. Despite this, it is possible that the mechanism for missing data was non-ignorable; in other words, the missing data depended on variables not measured in this study or on the health status of non-respondents at follow-up. We believe that the method of multiple imputation was adequate given the strong correlations between the variables that were used in the multiple imputation model. In addition, a necessary condition for this method was fulfilled [5]; the existing correlations between all factors used in the imputation model were systematically in the same direction. In this study, multiple imputation gives insight into the magnitude of selection bias on the prevalence estimates. Multiple imputation has some additional advantages above other methods to handle missing data; with multiple imputation all available information is used, therefore avoiding the loss of power associated with complete case analysis [5,6]. Furthermore, the fact that standard errors and confidence intervals resulting from multiple imputations are more appropriate than those resulting from other techniques such as single imputation is another important advantage of multiple imputation [5-7].

Besides non-response at follow-up, selective participation occurred at wave 1 of the longitudinal study in which 35.2% of all affected residents participated. Affected residents who participated at wave 1 were more likely to be women, to be between 25–44 or 45–64 years of age, to live with a partner, to be a single parent and to be of immigrant background. Analyses of multiple imputations showed that the selective participation did not affect the prevalence estimates of health problems at wave 1 [38]. In the present study we were, however, primarily interested in bias resulting from selective attrition at the follow-up surveys. Despite selective response, the prevalence estimates of health problems at wave 3 were not completely different when we corrected for the selective response by means of multiple imputation. This is important information, emphasizing the fact that selective response is only problematic when it biases the prevalence estimates of health problems.

## Conclusion

To date, most studies that have examined response in longitudinal studies have focused on whether respondents were systematically different from non-respondents. Our results indicate that considerable attrition and selective response only somewhat biased that prevalence estimates of health problems among survivors. Therefore, future studies, both among survivors of disasters and among the general population, should not only examine selective response, but should also investigate whether selective response has biased the prevalence estimates of health problems by using statistical techniques such as multiple imputation. Although the present study focused on potential bias in the prevalence estimates of health problems, investigations into risk factors for health problems should also take into account possible bias due to selective response. This is especially important in longitudinal studies after disasters since these studies examine the health needs of survivors and provide information on which post-disaster health interventions are based.

## Abbreviations

PTSD – Posttraumatic stress disorder

SCL-90 – Symptom checklist (90 items)

IES – Impact of event scale

VOEG – Questionnaire on subjective health complaints

RAND-36 – Questionnaire on general health (36 items)

OR – Odds Ratio

CI – Confidence interval

MCMC – Markov chain Monte Carlo

## Competing interests

The author(s) declare that they have no competing interests.

## Authors' contributions

BvdB performed the statistical analyses and was the principal writer of the paper. LG helped with the interpretation of the data and the writing of the paper. RKS was involved in the statistical analysis and the interpretation of the data. PvdV critically reviewed the manuscript. RKS, PvdV, and LG were involved in the acquisition of the data, conception, and design of the longitudinal study after the disaster. All authors read and approved the final manuscript.

## Pre-publication history

The pre-publication history for this paper can be accessed here:


